# A journey towards pediatric gastrointestinal endoscopy and its training: a narrative review

**DOI:** 10.3389/fped.2023.1201593

**Published:** 2023-07-28

**Authors:** Luca Scarallo, Giusy Russo, Sara Renzo, Paolo Lionetti, Salvatore Oliva

**Affiliations:** ^1^ Gastroenterology and Nutrition Unit, Meyer Children Hospital IRCCS, Florence, Italy; ^2^ Department of NEUROFARBA, University of Florence, Florence, Italy; ^3^ Pediatric Gastroenterology and Liver Unit, Department of Maternal Infantile and Urological Sciences, Sapienza University of Rome, Rome, Italy

**Keywords:** pediatric endoscopy, training in pediatric endoscopy, competency-based training, training the trainers, assessment of competence

## Abstract

**Background and aims:**

Gastrointestinal (GI) endoscopy in pediatric setting has unique features and, therefore, requires an approach that is tailored to pediatric practice. There is still heterogeneity between training programs worldwide in terms of duration, number of procedures and assessment during and at the end of the training process.

**Methods:**

We conducted a narrative review aiming to describe and summarize the existing literature on the various training methods for pediatric GI endoscopy to highlight the significance of specific pediatric endoscopy training.

**Results:**

Simulation-based tools have been implemented in several training programs, providing a safer learning environment for trainees, especially in their earlier stages of training. Assessment of competence is gradually shifting from the sole evaluation of procedural numbers towards the development of more reliable and valid tools that can accurately measure technical competence. Despite such seismic shift, there is still a need for a standardized and comprehensive pediatric-oriented endoscopy curriculum that incorporates acquisition of procedural skills education and is built on the current competency-based model of training. All the above must sink their roots in trainees and to ensure that the endoscopists of tomorrow are capable of delivering high quality of care for children undergoing endoscopy.

**Conclusion:**

It is crucial to parallelly focus on the way trainers teach trainees. In this context, the implementation of “train the trainers” courses has improved important quality meters in GI endoscopy. Future research should put the focus on the potential subsequent favorable benefits of these changes on child health.

## 1. Introduction

Gastrointestinal (GI) endoscopy is an essential part of training in both adult and pediatric gastroenterology. Indeed, the ability to safely and competently perform endoscopic procedures is a critical feature of GI practice (1). However, pediatric endoscopy has unique characteristics. For instance, the indications, the need and the use of anesthesia, the importance of routine tissue sampling and, last but not least, the emotional burden of performing an invasive procedure in children differ significantly from that of endoscopy in adults (2). Additionally, pediatric colonoscopy is rarely performed for colorectal cancer screening, the context that has guided the development of most current endoscopy quality metrics, including cecal intubation rates and adenoma detection rates (3). Therefore, pediatric endoscopy should preferably be performed by pediatric gastroenterologists worldwide (4, 5). During their training, pediatric gastroenterologists are expected to achieve endoscopic competency, defined as the minimum level of knowledge, skill, and expertise required to safely and competently perform endoscopy without assistance or supervision (1). Training in pediatric GI endoscopy continues to be based primarily on a trainer-apprentice model with most trainees learning basic endoscopy skills under the supervision of experienced endoscopists. Because adult endoscopy services typically perform a large number of procedures, trainee in pediatric endoscopy often draw on the experience of adult centers to implement their expertise (6). In addition, in some regions of the world, pediatric endoscopy can be performed by gastroenterologists trained to perform procedures on adults, as well as by other specialists, like pediatric surgeons (7). There is still a great deal of heterogeneity between training programs in terms of duration, number of procedures and assessment during and at the end of the training. Recently the Pediatric Endoscopy Quality Improvement Network (PEnQuIN) has addressed these criticisms to outline international standards for pediatric endoscopists and trainees, and the indicators that can be used to measure the quality of individual providers (8). Despite the consensus reached on a number of key standards that pediatric endoscopists should adhere worldwide, training in pediatric endoscopy is still widely unstructured.

The aim of this narrative review was to evaluate the existing literature on various training methods for pediatric GI endoscopy, including widely available training aids that have been developed to improve endoscopy education, and also to gather a comprehensive review on the assessment methods. The ultimate objective is to highlight the significance of pediatric endoscopy training in driving quality enhancements for pediatric endoscopy practice across the globe.

### 1.1. Methods

We conducted a comprehensive literature search to identify studies related to pediatric endoscopy training and quality improvement. We searched electronic databases, including PubMed, Scopus, and Web of Science, for articles published between January 2000 and March 2023. The main reason was for this choice was the paucity of data before the year 2000. The search strategy included a combination of keywords and Medical Subject Headings (MeSH) terms related to pediatric endoscopy training, education, quality improvement, and patient outcomes. We used “pediatric”, “children”, “gastrointestinal endoscopy”, “training”, “competence”, “skill acquisition” “assessment”, “simulation” as keywords. The search was limited to studies published in English. Two reviewers independently screened the titles and abstracts of the identified studies for relevance to the review topic. The full text of potentially relevant studies was then reviewed in detail to determine their eligibility for inclusion in the review. Inclusion criteria for the studies were: (a) studies that reported on training strategies, education programs, or quality improvement initiatives in pediatric endoscopy; (b) studies that assessed the impact of these interventions on patient outcomes or provider performance; and (c) studies that were published in English. Exclusion criteria were: (a) studies that focused on adult endoscopy; studies reporting mixed pediatric/adult results were included; (b) studies that did not report on training or quality improvement interventions; and (c) studies that were not published in English.

Two reviewers independently extracted data from the included studies, including study design, study population, intervention type, outcomes assessed, and results. Any discrepancies in data extraction were resolved by consensus or by a third reviewer if necessary. The data were synthesized narratively, with a focus on identifying common themes and trends in the literature related to pediatric endoscopy training and quality improvement.

The quality of the included studies was assessed using the Cochrane Risk of Bias tool for randomized controlled trials (9) and the ROBINS-I tool for non-randomized studies (10). Two reviewers independently assessed the quality of the studies, and any discrepancies were resolved through discussion or by a third reviewer if necessary.

As this study is a narrative review, no ethical approval was required. This narrative review was reported in accordance with the Preferred Reporting Items for Systematic Reviews and Meta-Analyses (PRISMA) statement for reporting systematic reviews and meta-analyses (11).

## 2. Skill acquisition: from apprenticeship models to competency-based training

The process of acquiring skills consists of three main consecutive phases (12). At the first stage, the trainee, beginner in practice, must focus only on fully understanding the procedure and minimizing possible mistakes. In this first phase, performance of the procedure is unreliable and the feedbacks from the trainer are mainly focused on explaining how the procedure is correctly performed and identifying common mistakes made by the learners. In the second phase, the trainee begins to translate the cognitive step learned in the first stage and to perform tasks more efficiently and fluently. Feedback is also essential in this second phase, as its absence is associated with no improvement in learner's skills (13). Finally, in the third and final phase, the procedures are automated, with little or no conscious awareness of the performance (12).

The gradual journey towards the abovementioned stages in order to acquire full competence is still largely based on the typical apprenticeship method, where trainees first observe the procedure, and then begin testing their hand. After all, the trainees should acquire a certain amount of specialist knowledge during the training period, which will enable them to carry out most of the procedures independently. The duration of this process depends on the length and intensity of the training as well as other individual factors. One of the main criticisms during the training process is that there is usually a lack of formal and objective in-training evaluation of progress. A typical scenario is that the trainer struggles with translating the skill into verbal input and overcomes the trainee's difficulties by taking over the scope.

In recent years, starting from adult's endoscopy centers and prompted by the implementation of national and regional colorectal cancer (CRC) screening programs, there has been a seismic shift in GI endoscopy training. To ensure high-quality and patient-centered endoscopy, quality indicators have been established, validated and monitored over time (14). A pivotal component of this major change was the recognition of the central role of endoscopic training models, moving from traditional methods to novel competency-based systems. Competence in GI endoscopy has been hard to delineate. In 1995, the American Society for Gastrointestinal Endoscopy (ASGE) defined competency in gastrointestinal endoscopy as “the minimum level of skill, knowledge, and/or expertise, derived through training and experience, required to safely and proficiently perform a task or procedure” (15). Skills have historically been grouped into two major domains: cognitive (i.e., understanding the basic elements of the procedure, knowledge of anatomy and physiology, obtaining informed consent and understanding sedation, and managing potential complications) and technical (i.e., handling the endoscope, advancing and/or strategies for mucosal inspection) (16). However, there is a third non-negligible domain represented by non-technical skills (i.e., patient assessment or organizational issues), that contribute to the achievement of favorable clinical and non-clinical outcomes (17). Despite the paucity of literature examining the impact of non-technical skills in pediatric endoscopy, literature deriving from adult care suggests they play a central role in quality care. *Exempli gratia*, a report of deaths occurring within 30 days of therapeutic endoscopy procedures, indicated that suboptimal teaching of non-technical skills (i.e., communication and/or teamwork) was more responsible for unfavorable outcomes than technical deficiencies (18).

## 3. Training aids for GI endoscopy: the role of simulation-based programs

### 3.1. Types of simulation devices

Typically, training in GI endoscopy is conducted in a “hands-on” setting, with a trainee learning to perform the procedure under the direct supervision of an experienced endoscopist. This model surely has some clear benefits since the one-to-one relationship with an experienced trainer provides the opportunity of immediate feedbacks during training. However, there are some obvious shortcomings in taking the first steps in GI endoscopy in a clinical setting and on actual patients. Indeed, it can be difficult to embrace an overload of information and feedbacks in a stressful environment, where the “trial and error” can increase discomfort and the risk of complications for the patients. In addition, time is a tyrant, and clinical demands can limit the trainer's ability to provide comprehensive directions and feedback during procedures. This issue is particularly relevant in the pediatric setting, as parents and trainers tend to be more protective of children, resulting in fewer cases available for trainees to work with (19). To address this challenge, integrating instructional aids into GI endoscopy training curricula could be a potential solution (20). Such an approach can provide a safe learning environment for trainees to develop their skills without harming patients, especially in the early stages of their learning. Simulation-based training is an effective way to achieve this goal, as it allows trainees to practice essential skills repeatedly, receive feedback from experts, and gradually improve their abilities (21). Over the last few decades, endoscopy simulators have been developed and improved continuously. These simulators can be broadly classified into four main types: mechanical models, *in vivo* and *ex vivo* animal models, and virtual reality (VR) simulators (22). While mechanical models were the first to be developed, live animal models are considered more realistic, but they come with significant drawbacks such as high costs, specialized facilities, deterioration over time, and ethical issues (23). To overcome some of these downsides, *ex vivo* models have been developed, combining animal organs with plastic parts. VR simulators, combining visual and haptic interfaces, present a wide range of scenarios resembling reality and/or tasks that are planned to train the learners in specific skills (24).

### 3.2. Simulation-based curriculum

Simulation-based learning programs have proven effective in supporting the classic apprenticeship model. A systematic review with meta-analysis of 21 randomized trials enrolling 1,181 participants by Singh et al. showed that simulation-based training in GI endoscopy significantly improved process skills and behaviors both in a test setting and in clinical practice. Additionally, it improved time to procedure completion and patient outcomes, such as procedural completion rate and risk of major complications (25). Another recent systematic review with meta-analysis focused on VR endoscopy simulation, included 18 randomized controlled trials with 421 participants and 3,817 endoscopic procedures. The study revealed that simulation-based endoscopic training was beneficial in complementing the performance of novice endoscopists before patient-based training, offering some competence-related benefits such as the rate of independent procedure completion, overall training performance and mucosa visualization (26).

There is limited research on the use of simulation-based training in pediatric endoscopy. Computer-based simulation training has been shown to improve trainees' confidence and technical skills, as measured by self-report assessments (27). However, a recent survey conducted among 71 pediatric GI fellowship program directors in North America and Canada highlighted that only slightly more than half of the programs integrate simulation-based training in their curricula, despite recognizing its importance, particularly for novices in GI endoscopy. The most commonly perceived barriers to its wider implementation in training programs were the equipment costs and the lack of a validated pediatric simulation curriculum (28). Providing the trainees with a simulation-based technology does not guarantee effective use. Trainers need to choose how to apply simulation-based learning to maximize its benefits for the novice endoscopists. Feedbacks is a crucial factor in procedural improvement, both in clinical and in a simulation settings (29). In the simulated setting, trainers can provide feedback at the end of task completion (terminal feedbacks) without concerns for patient safety, whereas this strategy is rarely feasible in the clinical setting. Walsh et al. found that the timing of feedback is a determinant of skill acquisition in the simulated setting (30). The authors reported that terminal feedback were more effective than feedback provided during task execution (30), as constant feedbacks may divert the trainee's focus from their performance to the feedback itself. Recent studies have focused on developing structured curricula for simulation-based endoscopy training. One such curriculum was designed by Grover and colleagues, which was divided into six hours of lectures and eighth hours of virtual simulator-based training with an expert tutor support. In a single-blinded randomized controlled trial, novice endoscopists assigned to the standardized curriculum demonstrated an improvement in technical, cognitive, and integrative skill achievement. They also exhibited an enhanced skill transfer into clinical setting, compared to those in a self-regulated learning group (31). Another study conducted a randomized controlled trial that included 39 novice endoscopists, who were assigned to either a control curriculum or a novel simulation-based curriculum that included dedicated non-technical skill (NTS) training. The NTS group outperformed the control group during two clinical colonoscopies, on the VR simulator, and in the integrated scenarios. The authors concluded that incorporating NTS training in simulation-based curricula is crucial for enhancing the overall performance of novice endoscopists (32).

To optimize the benefits of simulation-based training for pediatric GI endoscopy, it is essential to have a structured curriculum that appropriately challenges trainees. Grover and colleagues reported that a progressive simulation-based curriculum resulted in superior technical and non-technical skills, as well as an improved transfer to clinical setting (33). The findings emphasize the importance of matching the difficulty of tasks with trainee's ability to enhance the learning process. Additionally, less-technologically-advanced and less-expensive simulator may be optimal for basic skills training, reducing the financial burden of implementing simulation-based training in GI endoscopy.

Simulation-based training has proven beneficial, especially in the earliest phase of training. It helps accelerate learning curves by allowing novice endoscopists to become familiar with the endoscopic equipment, practice skills deliberately, and receive prompt, comprehensive feedback from an expert trainer without any harm to the patient (34). These features are particularly advantageous for pediatric GI endoscopy training, where even tertiary centers may have lower patient volumes, and there are greater concerns about taking the first steps in endoscopy with pediatric patients. However, efforts are still needed to enrich the literature with high-quality, specific pediatric evidence and to develop a standardized curriculum focused on the unique aspects of pediatric GI endoscopy.

## 4. Assessment

The shift towards competency-based curricula has highlighted the need for standardized assessment tools for evaluating GI endoscopy competence. Assessment is essential to optimize both learners' and the practitioners' capabilities, providing motivation and direction for future learning, protecting society from substandard care, and ensuring trainers have adequate competency before performing procedures independently (35, 36). Assessment can be broadly classified into two forms: formative and summative. Formative assessment aims to provide trainees with timely and comprehensive feedback, promoting self-reflection, stimulating and guiding future learning, and transitioning them from trainees to expert practitioners. Formative assessments can highlight specifc procedural strengths and weaknesses, allowing performance enhancing feedback and objective setting (37, 38). Additionally, formative feedbacks act to reinforce trainees' motivation to learn, they promote self-reflection, help students to identify their learning gaps, clarifies desired outcomes and encourages a dialogue about learning process (17). Conversely, summative assessment serves judgmental purposes, providing a comprehensive appraisal of outcomes such as competence, readiness for independent practice, and/or qualification for advancement. Summative assessments are used to provide self-regulation and accountability and must, therefore, have psychometric rigor. However, they can act as a barrier to further practice and learning for the trainee (35). The combination of formative and summative assessment is crucial in pediatric GI endoscopy learning process. During initial and subsequent practice, formative feedback can be used to enhance learning and to promote quality. In addition, summative assessments are required to ensure achievement and ongoing maintenance of competence. Assessment is an ongoing process that goes beyond the training period, a thoughtful integration of formative and summative assessment is pivotal to ensure effective learning and continued development of expertise. Standardized assessment tools for GI endoscopy competence have been developed and validated, but their adoption has been slow. To ensure their widespread use, these tools must be easily accessible, user-friendly, and integrated into the training curriculum. Regular assessment should be a part of the continuing professional development of practitioners to ensure that their skills remain up-to-date and to identify areas that require improvement.

### 4.1. Moving from procedural numbers to competence thresholds

Historically, competency in gastroenterology has been measured by the number of procedures a trainee has performed. However, these thresholds have generally based on expert opinion and may not accurately reflect a trainee's operative ability (39, 40). Furthermore, logbooks that record clinical experience have also been criticized for their reliance on procedural volumes, which can vary significantly among trainees and do not necessarily indicate technical competence (41). To address these issues, some guidelines have proposed the concept of a “competence threshold”, which is the number of supervised procedures required before a trainee's technical competence can be reliably assessed (4). Cass et al. reported an 80% success rate in esophageal intubation after completing 100 procedures (42), while another study showed 140 colonoscopies were required to reach a 90% rate of cecal intubation (43). However, more recent studies have suggested that these numbers may be higher, with some estimating that over 300 colonoscopies are required to reach a 90% technical success rate (44, 45). Establishing competence threshold for pediatric GI is particularly challenging, given the smaller number of endoscopies performed in children and the lack of high-quality literature on learning curves for pediatric endoscopists. Discrepancies have been observed among the guidelines that establish the competence threshold for both pediatric GI endoscopy since they have been largely extrapolated data from adult studies (19). Table 1 summarizes the competence thresholds as defined by different adult and pediatric GI scientific societies.

**Table 1:** Procedural numbers and other requirements for GI endoscopy according to different pediatric and adult scientific societies.

Scientific organization	Country	Upper gastrointestinal endoscopy		Lower gastrointestinal endoscopy	
		Competence threshold	Other requirements	Competence threshold	Other requirements
Pediatric					
ESPGHAN (4)	*Europe*	Not specified	Not specifically defined	Not specified	Not specifically defined
NASPGHAN (5)	*North America*	100	10 foreign body removals	120	10 snare polypectomies
			15 with control of bleeding with various methods and/or colonos- copy with control of bleeding		≥90% cecal intubation rate by the end of fellowship
Conjoint committee for recognition of training in gastrointestinal endoscopy (46)	*Australia*	200	≥100 in pediatric patients, ≥10 therapeutic procedures of which ≥5 control of bleeding	100 (≥75 in pediatric patients, “some polypectomy experience”)	≥90% cecal intubation rate
					≥10 therapeutic procedures of which ≥5 involve control of upper GI hemorrhage supervision of recognized pediatric supervisor
BSPGHAN (47)	*UK*	100	Intubation of D2 >95%	100	≥ 60% independent cecal intubation rate cecal intubation >90%
			Retroflexion >95%		
			Unassisted physically >95%		
			DOPS >90% (rated as competent)		
					DOPS >90% (rated as competent)
			Attended “Basic Skills” Course in upper GI endoscopy		
					Serious complications <0.5%
			Summative assessment (≥2 assessors ≥ procedures)		
					Attended “Basic Skills” Course in lower GI endoscopy
					Summative assessment (≥2 assessors ≥ procedures)
PEnQuIN (8)	*Europe & North America*	N/A	Achievement of competence to perform specified routine and/or emergency pediatric procedures according to appropriate current standards.	N/A	Achievement of competence to perform specified routine and/or emergency pediatric procedures according to appropriate current standards.
					Unadjusted cecal intubation rate ≥90%
					Unadjusted terminal ileal intubation rate ≥85%
Adult					
European diploma of gastroenterology (48)	*Europe*	300		100	
ASGE (49)	*North America*	130		140	
SAGES (50)	*North America*	35		50	
Korean society of gastrointestinal endoscopy (51)	*South Korea*	1,000		150	
BSG (52)	*UK*	250	D2 intubation rate ≥95%	280 (or 200 if certified for flexible sigmoidoscopy)	Unassisted Caecal Intubation Rate ≥90%
			J-manoeuvre ≥95%		
			Unassisted physically ≥95%		
			Attended “Basic Skills” Course in upper GI endoscopy		
					Unassisted terminal ileal intubation rate (in patients with suspected IBD, e.g. anaemia and chronic diarrhoea) ≥60%
			To complete DOPS throughout training, 1 DOPS form for every 10 procedures		
			DOPs >90% (rated as competent)		
					Attended “Basic Skills” Course in lower GI endoscopy
					DOPs >90% (rated as competent)

ESPGHAN, European Society of Pediatric Gastroenterology Hepatology and Nutrition; NASPGHAN, North American Society of Pediatric Gastroenterology Hepatology and Nutrition; BSPGHAN, British Society of Pediatric Gastroenterology Hepatology and Nutrition. PEnQuIN, Pediatric Endoscopy Quality Improvement Network; ASGE, American Society for Gastrointestinal Endoscopy; SAGES, Society of American Gastrointestinal and Endoscopic Surgeons; BSG, British Society of Gastroenterology.

Additionally, obtaining the recommended number of specialized procedures required to meet competency standards may be difficult, even in tertiary facilities (53, 54). In a survey of 50 third-year pediatric GI fellows, only slightly more than half had performed more than 100 colonoscopies (55). Despite these challenges, it is crucial to establish competence thresholds to optimize learning and ensure patient safety. However, more research is needed to determine appropriate thresholds for pediatric GI endoscopy, as well as to develop reliable and valid assessment tools that can accurately measure technical competence.

### 4.2. Quality metrics

Broekaert et al. recently conducted a survey conducted among young members of the European Society for Pediatric Gastroenterology, Hepatology and Nutrition (ESPGHAN) to evaluate the quality of pediatric endoscopy training across European centers. Despite completing a median of 200 upper GI endoscopies and 75 colonoscopies (both above the threshold suggested by the ESPGHAN syllabus), only 43% of the 68 surveyed trainees achieved a terminal ileum intubation rate >90%, highlighting the need of a more rigorous and standardized assessment of competency (56). The recent focus on optimizing quality in endoscopy has influenced the assessment of competency, with many scientific societies incorporating quality metrics in their credentialing guidelines (Table 1). However, while quality metrics may be a useful indicator of trainee's overall performance, they do not provide continuous feedback or outline criticisms or deficiencies. Adenoma detection rate applies poorly to pediatric GI endoscopy, since colonoscopies in children are infrequently performed with cancer screening purposes. In contrast, terminal ileum intubation rate is more important when in pediatric colonoscopy, as colonoscopy is often performed to diagnose inflammatory bowel disease, which requires complete ileal intubation, exploration, and sampling (57). The recently published guidelines from the Pediatric Endoscopy Quality Improvement Network (PENQUIN) working group set minimum targets for defining high quality ileocolonoscopy as an unadjusted cecal intubation rate of ≥90% and an unadjusted terminal ileum intubation rate of ≥85% (3, 8).

### 4.3. Competence assessment tools

The completion of a certain number of supervised procedures does not guarantee that a trainee is adequately prepared to perform GI endoscopy independently. Rather, these numbers can be used as an adjunct to the more focused formative assessment. To document progress and proficiency level during endoscopy training, the recently published joint NASPGHAN/ESPGHAN guidelines from the Pediatric Endoscopy Quality Improvement Network working group recommend the use of competence assessment tools with strong validity evidence (8). However, there is currently limited literature on whether the implementation of these tools can improve clinical outcomes. Despite this, the Pediatric Endoscopy Quality Improvement Network working group has reached a consensus on the routine use of certain well-validated tools for the assessment of competence during GI endoscopy training. One of these tools is the direct observation of the procedural skills (DOPS) which is well-established among adult practitioners in the UK. DOPS assessments are performed by a trainer observing a trainee and are submitted electronically to the Joint Advisory Group on Gastrointestinal Endoscopy Electronic Training System e- Portfolio (37, 52, 58). Recent studies have provided low-quality evidence to support the use of DOPS scores to describe both technical and non-technical competency during pediatric upper GI endoscopy and ileocolonoscopy with sufficient sensitivity and specificity (59, 60). However, the only tool specifically developed for the assessment of pediatric ileocolonoscopy is the Gastrointestinal Endoscopy Competency Assessment Tool for pediatric colonoscopy (GiECAT_KIDS_) (61). This tool was developed through a Delphi process by 41 North American pediatric endoscopy experts who established key colonoscopy aspects for proficiency acquisition. After item reduction and gradation, 18 checklist and 7 global rating items were generated, reflective of technical, cognitive, and integrative competency required for safe and proficient endoscopy (61). The same group validated GiECATKIDS by assessing 104 colonoscopies from 56 endoscopists across 3 North American centers, demonstrating the tool's strong reliability and validity as a measure of pediatric colonoscopy performance (62). Despite this rigorous development and validation process, the tool is not widely utilized.

## 5. Training the trainers

**Figure 1:**
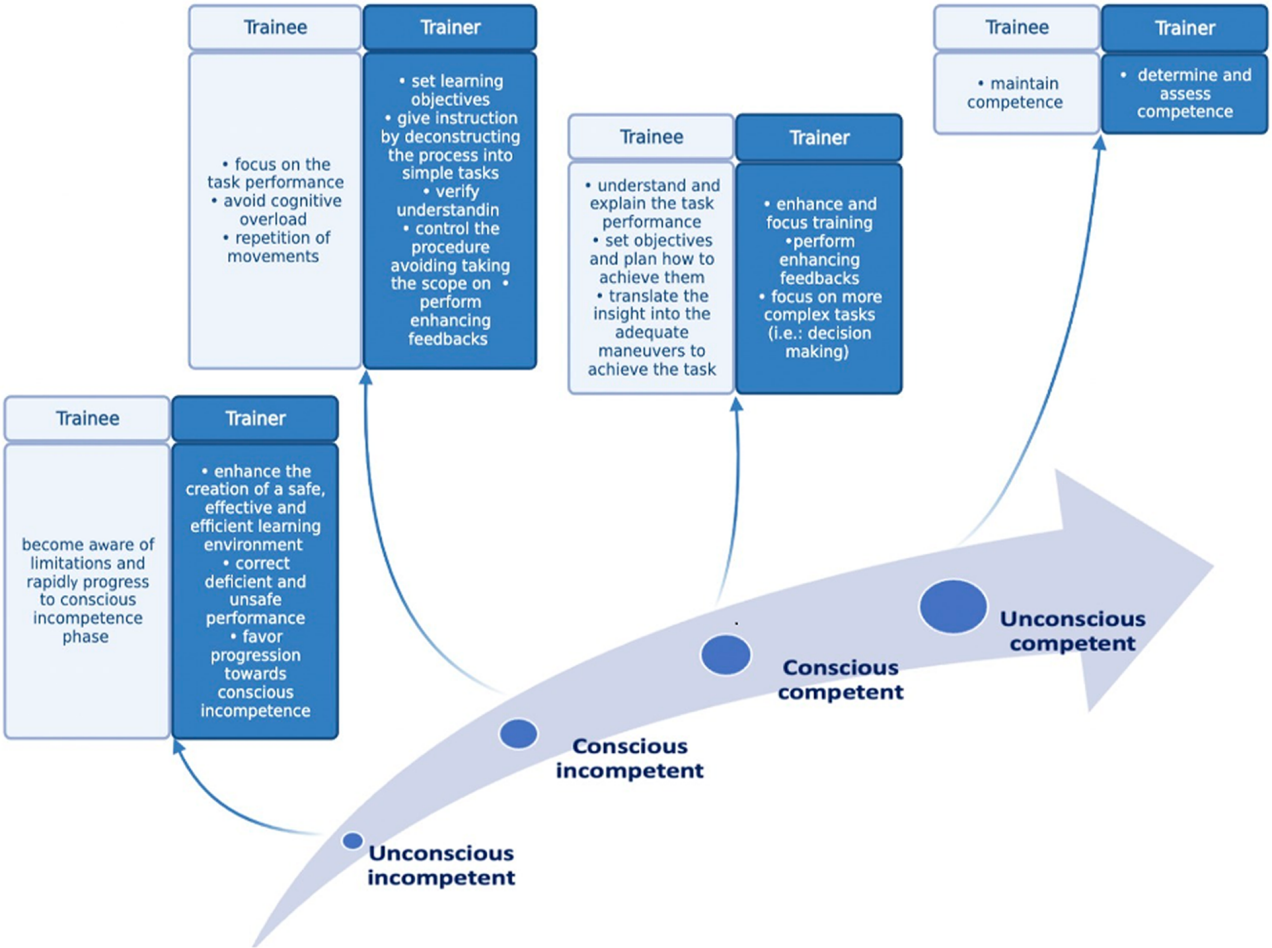
Steps for achievement and maintenance of competence in delivering pediatric endoscopy care.

Competency-based training involves both trainees and trainers. Despite ongoing efforts to structure a standardized training program for pediatric GI endoscopy, it is surprising that most of the endoscopy training is provided by endoscopists who have not had any education addressing how to teach endoscopy. Unfortunately, this is not surprising given the lack of literature addressing this specific issue. However, the implementation of cancer screening programs worldwide has drawn attention to the need of higher quality in delivering endoscopy training. In the UK, a “train the trainer” course module has been implemented for faculty who teach gastrointestinal endoscopy (63) and subsequent implementation has shown improvement in colonoscopy quality outcomes in the UK (63). The train the trainer or train the colonoscopy trainer (TCT) courses have since been shared with other health systems. The central concept in TCT courses is how skill acquisition occurs. According to one of the most accredited models for skill acquisition, trainees during the skills acquisition process progresses through 4 main stages: (i) unconscious incompetence, where novices are completely unaware of what they don't know; (ii) conscious incompetence, where they become cognizant of what they don't know; after the first two phases the individual starts to gain proficiency in the procedure and becomes able to perform them without paying too much attention to them (phase of unconscious competence). If a given task is learned without having all its parts rehearsed, the trainees bypass the (iii) “conscious competence” phase and progress directly to the (iv) “unconscious competence” one. With insightful training aimed at achieving conscious awareness of endoscopy skills, the learner achieves a conscious competence stage. In this stage, the trainer is able to understand and explain what needs to be done and is able to translate that insight into the adequate maneuvers to achieve the task. Subsequently, over time, the learner progresses to a stage of automatic learned movements performed without having to think about each component of the movement. Therefore, most expert endoscopists tend to be unconsciously competent, after having lost their awareness over time of after having become unconsciously competent without a proper conscious phase. However, to be an effective trainer, endoscopist need to have achieved and maintained a phase of conscious competence. Indeed, the ability to deconstruct a complex task in simpler parts and to analyze each of them individually is critical to teach others efficiently. Moreover, trainers should be able to guide trainees through procedural challenges without taking over the scope in order to enhance trainee's self-confidence (Figure 1). Thus, TCT courses aim to train faculty to achieve conscious competence (64). Implementation of TCT courses has shown direct clinical benefits for patients. For instance, in a randomized controlled trial from Poland, Kaminski and colleagues demonstrated that faculty assigned to a Train-Colonoscopy-Leaders (TCLs) program improved important quality measures in screening colonoscopy (i.e., the adenoma detection rate) among colonoscopy screening centers with suboptimal performance (65). Future research should focus on the development of specific pediatric-focused TCT courses to improve the quality of pediatric endoscopy training.

## 6. Limitations

The present work has several limitations, most of them are intrinsically related to the narrative nature of our review. We thoroughly reviewed the literature, but we didn't conduct any pooled analysis of the data that we have summarized. However, as previously mentioned in the review, pediatric GI endoscopy training programs, as well as the number of procedures required to achieve “competency” and assessment during and at the end of the training process are highly heterogeneous. This heterogenicity makes harder to perform a systematic review and its conclusions less generalizable.

## 7. Conclusions

Delivering high-quality training is crucial to provide safe, efficient, and effective care in pediatric GI endoscopy. Pediatric endoscopy is a complex skill and can be challenging to learn, but also to teach. It is well-established that performance of endoscopy in children requires pediatric-specific training and assessment. The rigorous process undertaken by the PEnQuIn working group in developing a list of key quality standard that should be upheld by all pediatric endoscopists and endoscopists in training is a cornerstone aimed at raising the quality of care for children undergoing endoscopy. It is self-explanatory that to improve quality in pediatric GI endoscopy it is essential to focus on high-quality training for endoscopists. The ideological movement that shifted the focus of pediatric GI endoscopy on patients on their families must sink its roots in the trainees, the endoscopists of tomorrow. This requires a continuous effort by the endoscopic community to ensure that endoscopic training is adequately supported (time, facilities, and funding allocation), evidence-based and efficient, regardless of its location. Therefore, it is crucial to also focus on the way trainers teach trainees, which can significantly improve the overall quality of pediatric endoscopy. Future research should also put the focus on training programs implementation and on the potential subsequent favorable benefits on child health.
